# Fostering resilience during COVID-19: a salutogenic framework for managing disruptive change

**DOI:** 10.3389/fpsyg.2026.1879475

**Published:** 2026-09-21

**Authors:** Antoni Barnard

**Affiliations:** Department of Industrial and Organizational Psychology, University of South Africa, Pretoria, South Africa

**Keywords:** COVID-19, hermeneutic phenomenology, holding environment, resilience, retail employees, salutogenic, sense of coherence

## Abstract

COVID-19 presented a poignant learning opportunity for managing and supporting employees’ well-being in work contexts affected by disruptive change and unforeseen disaster events. The retail sector was particularly challenged to maintain a resilient workforce during this time. Employees worked on the frontline without having the training or skills like those of health workers, to cope with the psychological demands incurred by working face-to-face with the public. To glean insights into organizational and management practices that foster resilience during times of disruptive change, this study explored the experiences of retail employees who were exposed to the height of the COVID-19 pandemic in South Africa. The study employed a hermeneutic phenomenological approach, conducting semi-structured interviews with 27 employees who were actively working in a South African retail organization during the country’s first (May to July 2020; *n* = 11) and second (January to April 2021; *n* = 16) COVID-19 waves. Thematic analysis was informed by salutogenic theory, with resilience and sense of coherence as key guiding constructs. Addressing the lack of research on how organizations can foster resilience, the findings suggest salutogenic practices for managing disruptive change in the retail work context. Practices are recommended that can: (i) establish coherence and support comprehensibility, (ii) restore control and support manageability, and (iii) nurture connectedness and support meaningfulness. The study contributes to existing knowledge by linking resilience to sense of coherence in a pragmatic salutogenic framework, that describes the organization as a holding environment for coping with disturbing emotions and making sense of difficult circumstances.

## 1 Introduction

The COVID-19 pandemic, and subsequent lockdown regulations (2020–2022), disrupted the workplace and heightened employees’ uncertainty, anxiety and fear (Damghanian et al., 2023). Employees in the retail sector notably experienced escalated levels of anxiety and depression, with the industry being affected by store closures, economic uncertainty, customer challenges and employee health risks (Benchekroun et al., 2022; Choi and Marinescu, 2025). At the time, research focused predominantly on healthcare workers, first responders, medical students and the general population (Chen et al., 2021; Mayer et al., 2022; Seaborn et al., 2022) with Voorhees et al. (2020) aptly referring to retail workers as the *forgotten frontline*.

Rosemberg et al. (2021) portray retail workers as having been distinctly vulnerable and fragile during the COVID-19 pandemic. When most blue-collar workers started working from the safety of their homes, brick-and-mortar retail employees suddenly became the indispensable frontline workers having to contend with significant health and safety stressors for which they were untrained and had no prior experience (Ali Sulaiman et al., 2020; Blau et al., 2021; Elnahla and Neilson, 2021; Jacobson et al., 2022; Mayer et al., 2022). Direct customer contact heightened retail workers’ anxiety and stress because social distancing was difficult to maintain, and customer interactions were complicated by elevated levels of public anxiety. These aspects augmented the psychological demands and mental workload of retail employees, increasing their risk for anxiety, depression, post-traumatic stress and burnout (Choi and Marinescu, 2025; Janson et al., 2021; Rodríguez-López et al., 2021; Swazan and Youn, 2023; Voorhees et al., 2020). Retail work consequently became a high-risk occupation at the time (Barbieri et al., 2022; St-Denis, 2020). Key phenomena observed in the retail environment were increased workload, existential fear and survival anxiety, job and economic insecurity, volatile and demanding customer interactions, feelings of disempowerment, lack of management support and recognition, and increased work–family conflict (Elahi et al., 2022; Elnahla and Neilson, 2021; Gaan and Shin, 2023; May et al., 2021; Rosemberg et al., 2021; Swazan and Youn, 2023; Voorhees et al., 2020). The psychological impact of COVID-19 on retail workers and limited research in this regard highlight the need for studies that inform the support and management of these workers during crises and catastrophic events (Damghanian et al., 2023; Gaan and Shin, 2023; Janson et al., 2021; May et al., 2021; Mayer et al., 2022; Voorhees et al., 2020). Knowledge about how to support and manage employees’ coping and resilience during periods of disruptive change is essential since subsequent mental distress can undermine motivation and heighten workplace attendance anxiety (Choi and Marinescu, 2025) and lead to poor organizational citizenship behaviors and a decline in employee integrity (Mayer et al., 2022; Swazan and Youn, 2023).

Organizations were ill-prepared to address the psychological effects of the pandemic on employee well-being, particularly in terms of supporting employees’ coping and resilience during this time (Azizi et al., 2021; Carnevale and Hatak, 2020; Hamouche, 2023). Retail organizations, in particular, encountered unique challenges in sustaining employee motivation, mental health and productivity (Ali Sulaiman et al., 2020). Studies noted the detrimental effects that unsupportive employers (Choi and Marinescu, 2025) and strained supervisor relationships (Gaan and Shin, 2023) had on the mental well-being of retail workers during the pandemic. However, explicit research focusing on the management of operations and employees during the COVID-19 crisis is sparse (Hamouche, 2023). Research focused mainly on managing remote or virtual workers (Bartsch et al., 2021; Lee, 2021) and healthcare workers (May et al., 2021; Mayer et al., 2022; Voorhees et al., 2020). The limited body of research that explored the experiences of retail workers during the pandemic focused on specific populations, such as part-time retail employees (Choi and Marinescu, 2025) and aimed to identify particular stressors in retail (Elnahla and Neilson, 2021) such as job insecurity (Ghani et al., 2022), customer conflict (Mayer et al., 2022; Northington et al., 2021) and perceived workplace safety (Mayer et al., 2022). Other investigations have sought to predict or describe the adverse effects of COVID-19 on burnout (Rodríguez-López et al., 2021) and general mental well-being (Janson et al., 2021; Mayer et al., 2022; Rosemberg et al., 2021).

Implications for organizational and management support practices are informed by insights derived from research identifying stressors as well as from conceptual reviews (Asongu and Rahman, 2022; Carnevale and Hatak, 2020) and secondary sources (Ali Sulaiman et al., 2020). There seems to be a gap in COVID-19 research focusing explicitly on examining and consolidating organizational support practices in the retail sector, and even more so from a salutogenic perspective. COVID-19 literature highlights the scarcity of and need for resilience research on retail employees (Asthana et al., 2024; Karanikas et al., 2023). As retail employees were thrust into high-risk frontline roles during COVID-19 without adequate preparation, increasing their psychological vulnerability, it is essential to examine practices that support coping and resilience to reduce distress, sustain productivity, and strengthen the retail sector’s preparedness and socio-economic stability in future crises. Moreover, examining management practices in this pertinent context may contribute to broader workplace resilience theory, particularly as the conceptual multiplicity of resilience complicates its operationalization (Smuda-Kocoń, 2024). Therefore, the aim of this study was to describe organizational and management practices that enable retail employees to cope and remain resilient under challenging circumstances within a unified framework that is theoretically grounded and pragmatically useful. To achieve this aim, the following research question directed the research: How do organizational and management practices in a retail organization support employee coping and resilience during disruptive change such as the COVID-19 pandemic, and what insights do these practices offer for managing employees in high-risk circumstances and for the advancement of resilience theory? The study entailed an exploratory and descriptive qualitative inquiry, conducted from a hermeneutic phenomenological perspective. Salutogenic theory was applied as interpretive lens guiding analysis to focus on what went right, rather than what went wrong (Louw and Mayer, 2014).

The paper is structured as follows: First, the theoretical framework guiding the study is presented, after which the research approach and methods are discussed. The findings are presented, and the paper concludes with a discussion of these findings, limitations of the study and recommendations for practice and future research.

## 2 Theoretical framework

Salutogenesis, a positive psychology approach (Louw and Mayer, 2014) conceptualizes health and illness as opposite ends of a continuum, rather than separate states (Forooraghi et al., 2022). Coined by Antonovsky (1987) it emphasizes factors that promote health rather than those that cause illness. In response to calls for increased visibility of salutogenic approaches in work-related research and practice (Jenny et al., 2022; Mana et al., 2021; Sethuraman, 2020), the study’s theoretical framework was guided by salutogenic constructs, specifically resilience and sense of coherence (SOC).

### 2.1 Resilience

Resilience, defined initially as an individual’s capacity to adjust to adversity (Burnard and Bhamra, 2019), has since evolved to encompass systems and process-based perspectives. The systems perspective acknowledges that resilience is impacted by both external and internal influences and operates across multiple systemic levels, including the individual, group and organizational domains (Galy et al., 2023). The process perspective recognizes resilience not only as a reactive coping resource but also as a dynamic developmental capacity through which individuals and organizations can strengthen and grow from adverse circumstances (Burnard and Bhamra, 2019; Hartmann et al., 2020; Van Breda, 2018). Adaptation and recovery are thus recognized as the two core components of resilience within an organizational system (Galy et al., 2023; Wut et al., 2022). Organizational and individual resilience are interdependent constructs shaping each other (Ben-Hador and Yitshaki, 2025). Organizational resilience is defined as the organization’s capacity to respond constructively to disruptive and threatening circumstances (Wut et al., 2022) and to transform itself to support sustained functionality and long-term adaptability (Southwick et al., 2014). Individual resilience is conceptualized as psychological resilience, an internal, personal resource that enables individuals to withstand, recover from, and grow through stressors (Den Hartigh and Hill, 2022; Wut et al., 2022).

Developing and supporting employee resilience is key to organizational resilience, yet the measurement and operationalization of resilience remain challenging due to different resilience perspectives emphasizing different aspects of the concept (Den Hartigh and Hill, 2022; King et al., 2016; Sisto et al., 2019; Smuda-Kocoń, 2024; Vella and Pai, 2019). To support empirical engagement with the concept, psychological resilience has been framed within Antonovsky’s salutogenic paradigm (Antonovsky, 1987, 1996), which emphasizes positive strength-based constructs. Among these, SOC is often highlighted as a key resource for constructive coping and adaptive development in the face of adversity (Hartmann et al., 2020; Van Breda, 2018).

### 2.2 Sense of coherence

Sense of coherence is conceptualized as a resilience resource and a wellness-promoting life orientation, enabling individuals to perceive life circumstances as comprehensible, manageable and meaningful (Feldt et al., 2011; Roskams and Haynes, 2020). Sense of coherence supports resilient behavior in the workplace because it positively affects an employee’s capacity to cope with regular, acute or chronic stressors, thereby sustaining well-being and productivity (Forooraghi et al., 2022; Mana et al., 2021). The SOC sub-component of *comprehensibility* supports constructive coping as people cognitively make sense of their circumstances, perceiving them as structured, coherent and explainable. *Manageability* is the behavioral SOC component and entails a sense of having the needed resources to deal with challenges. *Meaningfulness*, the motivational component, supports coping because it enables individuals to perceive life’s challenges as worthy of engagement and exerting effort (Antonovsky, 1987; Feldt et al., 2011). Sense of coherence moderates the effect of adverse conditions on employee well-being (Broetje et al., 2020; Jenny et al., 2022). In the context of COVID-19, studies have highlighted SOC as a key factor in reducing stress and maintaining optimal functioning at work (Barni et al., 2020; Mana et al., 2021). Sense of coherence, in turn, is influenced by job resources and coherent work experiences (Broetje et al., 2020; Jenny et al., 2022; Roskams and Haynes, 2020), underscoring the relevance of Work-SOC, experiencing the work environment as comprehensible, manageable and meaningful (Bauer and Jenny, 2007). Organizations, through its structures, strategies and culture significantly impact employees’ salutogenic health (Forooraghi et al., 2022). By providing structure, consistency and routine, organizations constitute a holding environment that strengthens employees’ SOC (Rob, 2013). The organization as holding environment relates to the system psychodynamic notion of containment, which describes the organization’s ability to hold employees’ anxiety in such a way that they can make sense of difficult circumstances, thereby increasing their resilience (Eloquin, 2016; Kahn, 2001; Petriglieri and Petriglieri, 2010). A holding environment is characterized by safe, reliable workspaces that allow emotional expression and enable rational thought and action (Kahn, 2001).

Research indicates a positive correlation between resilience and SOC (Kase et al., 2024). Broadly resilience refers to a person’s capacity to adapt positively to adversity and SOC, reflecting an individual’s orientation to life as being comprehensible, manageable and meaningful, enables the capacity to adapt positively under duress. However, due to various resilience perspectives (Smuda-Kocoń, 2024), proposing a conceptual alignment between resilience and SOC requires choosing a specific conceptual framework to resilience. The 3-Cs resilience framework of Reich (2006) was chosen here, because it proposes a conceptual approach to enhance resilience during times of crisis. It has not been empirically validated but theoretically outlines three psychological principles for organizations to cultivate: coherence, control and connectedness. The 3-Cs framework is useful for crisis management such as the COVID-19 pandemic as it provides a theoretical framework for developing interventions aiming to make sense of dire experiences and cope with debilitating emotions of fear (Barni et al., 2020). Reich’s (2006) framework also conceptually aligns with SOC, proposing that a supportive work environment should (i) address uncertainty and confusion (related to comprehensibility) by enabling *coherence*, (ii) offer manageable options to cope with the crisis (related to manageability) and thus regain a sense of *control*, and (iii) build formal and informal relationships that are emotionally supportive and motivating (related to meaningfulness) to establish *connectedness* (Barni et al., 2020; Reich, 2006; Sethuraman, 2020). Figure 1 illustrates the proposed link between coherence, control, and connectedness (resilience constructs in Reich’s (2006) 3-Cs framework) and the SOC sub-components: comprehensibility, manageability and meaningfulness.

**Figure 1:**
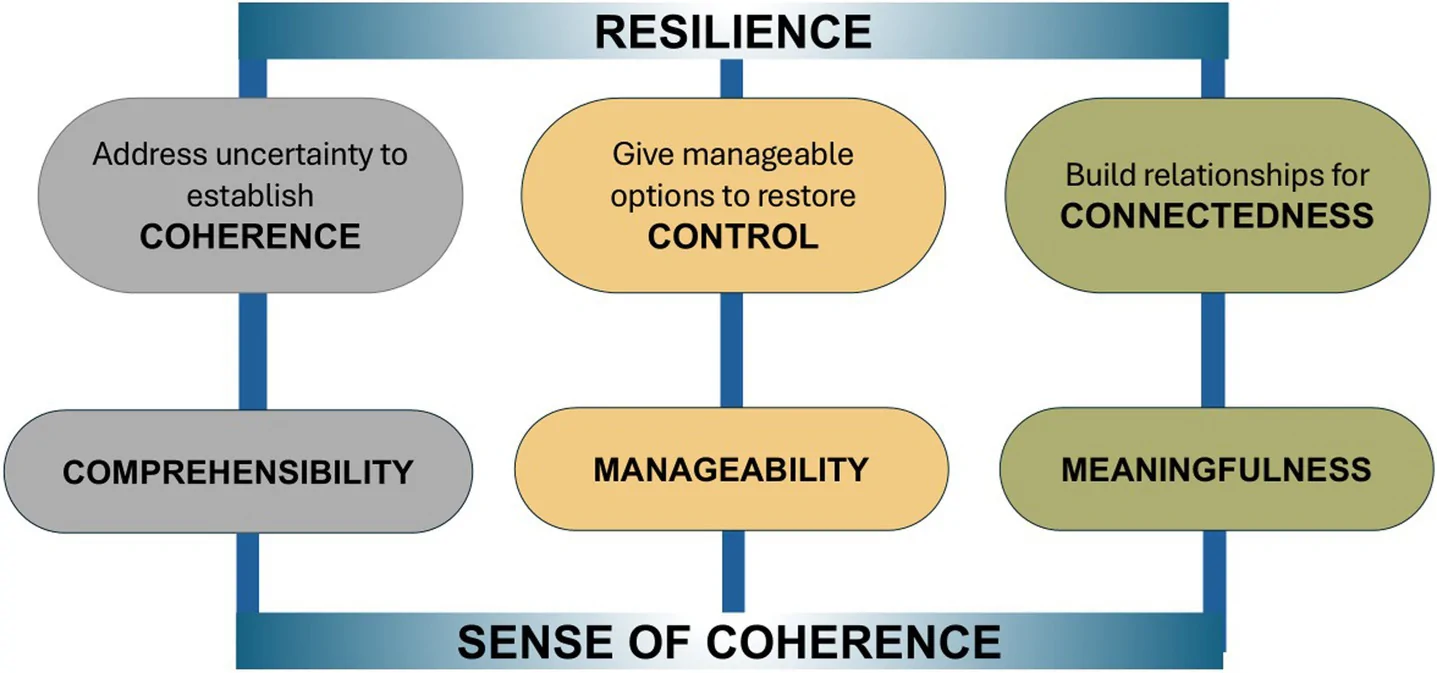
Linking resilience constructs with SOC sub-components.

## 3 Research design

### 3.1 Research orientation

The study is grounded in a hermeneutic phenomenological orientation and a qualitative research strategy. Hermeneutic phenomenology studies a phenomenon of interest by exploring people’s lived experiences and making meaning thereof through interpretive processes (Crowther and Thomson, 2020; Lindseth and Norberg, 2022). The approach was deemed appropriate to study people’s emotional and personal experience and how they made sense of the unique context of COVID-19. Furthermore, hermeneutic phenomenology allows for researcher interpretation and acknowledges the role of personal and theoretical predispositions. Consistent with a hermeneutic phenomenological stance, I recognize that I did not approach the study as a completely neutral or unbiased observer. While dealing with my own COVID-19 related emotions and work-life challenges, as a psychologist I was intrigued at the time with how other people were coping. In previous research I have demonstrated a preference for studying employee coping, sense of coherence and identity resilience in uniquely challenging work contexts. In this study knowledge is therefore constructed through a double hermeneutic process, which entails eliciting narratives of lived experiences that reveal participants’ understanding of their experiences and blends these with the researcher’s interpretations (Alsaigh and Coyne, 2021; Suddick et al., 2020). Semi-structured interviews were conducted, and thematic analysis, a popular hermeneutic-phenomenological strategy of analysis (Ho et al., 2017), was applied to construct meaning from the data.

### 3.2 Research context and procedure

The study was conducted in a large family-owned retail organization in South Africa following the country’s first COVID-19 wave (March to November 2020) and second wave (December 2020 to April 2021). At the start of the study, excluding part-time and temporary staff, the study population included 1,175 permanent staff working across eight stores and a head office. The South African government declared the COVID-19 pandemic a national disaster, imposing a national lockdown on 26 March 2020, when the company stopped operations and closed its stores. The government enforced an alert system with five restriction levels. Level 5 entailed the highest restrictions, confining citizens to their residences and restricting economic operations. When Level 4 commenced on 1 May 2020, allowing some business operations for the sale of essential goods, the company reopened its stores. A few staff members initially returned to work in shifts, but record sales from May to September prompted the return of all. An additional 240 employees had been appointed by the end of 2020 to accommodate the increased workload. Lockdown Levels 1 and 2 allowed face-to-face interaction under specific conditions, and 11 interviews were conducted between November and December 2020, at Level 1. An additional 16 face-to-face interviews were conducted between 14 May and 15 June 2021 at Level 2. By this time, the company had confirmed 90 positive employee cases, fortunately, with no loss of life.

### 3.3 Sampling and participants

Purposive sampling was employed, intentionally selecting participants who had experienced the research phenomenon and were willing to share their experiences (Alase, 2017; Ramsook, 2018). Participants were included based on their permanent employment with the company, active work during the first COVID-19 wave in 2020 and the second wave in 2021 and either having tested positive for the virus or having had close contact with someone who tested positive. Following the first wave of COVID-19 in 2020, 14 eligible employees were identified by the human resources manager (HRM), and a further 25 after the second wave in 2021. Willing participants’ contact details were provided to the researcher by the HRM, together with the role, store location and whether they had tested positive or had to isolate due to close personal contact. To limit further potential recruitment bias, the researcher privately contacted potential participants once lockdown levels were relaxed to Level 1 or 2, to arrange in-person interviews. Of the 39 identified employees, 12 were excluded because they were unreachable due to leave or work commitments. A few were also excluded because lockdown restrictions were increased again in mid-June 2021, which prevented in-person data collection. The final sample (*n* = 27) size considerations align with decision criteria such as the diversity of the staff population, the breadth and focus of the research question and pragmatic constraints (Braun and Clarke, 2021). The sample ultimately ensured good heterogeneity in the experiences shared, as it included frontline sales staff, support staff and managerial-level staff across various racial and gender groups at different stores. Data analysis furthermore provided adequately rich and varied thematic interpretations, allowing for thematic or interpretive saturation (Braun and Clarke, 2021). The 27 participants (11 after the first wave and 16 after the second wave), are detailed in Table 1. For confidentiality, participant codes are used: W1P1 refers to the interview with participant 1, conducted after the first COVID-19 wave, and W2P1 refers to participant 1 from interviews after the second COVID-19 wave.

**Table 1:** Participants.

Participant	Gender	Race	Job level	Frontline/Support	Covid-19 test result
W1P1	F	W	Junior management	Support	Positive
W1P2	M	B	Employee	Frontline	Positive
W1P3	M	W	Junior management	Frontline	Positive
W1P4	F	B	Employee	Support	Positive
W1P5	F	B	Employee	Frontline	Positive
W1P6	M	W	Employee	Frontline	Positive
W1P7	F	B	Employee	Frontline	Positive
W1P8	F	W	Junior management	Frontline	Positive
W1P9	F	W	Employee	Support	Positive
W1P10	F	B	Employee	Support	Positive
W1P11	F	W	Middle management	Frontline	Negative
W2P1	F	W	Employee	Support	Positive
W2P2	M	W	Employee	Support	Positive
W2P3	F	W	Employee	Frontline	Positive
W2P4	F	W	Junior management	Frontline	Positive
W2P5	F	B	Employee	Frontline	Positive
W2P6	M	B	Employee	Frontline	Positive
W2P7	M	B	Employee	Frontline	Positive
W2P8	M	B	Employee	Frontline	Positive
W2P9	M	B	Employee	Support	Positive
W2P10	F	B	Employee	Support	Positive
W2P11	F	B	Employee	Frontline	Positive
W2P12	F	W	Senior management	Support	Negative
W2P13	M	W	Junior management	Support	Positive
W2P14	M	W	Senior management	Support	Negative
W2P15	F	W	Senior management	Frontline	Negative
W2P16	M	W	Senior management	Frontline	Negative

Frontline employees work in direct contact with customers on a daily basis. Support staff do not have daily contact with customers, but they may occasionally interact with them. Support staff returned to work at retail stores, where they worked, for example, in HR, Buying, Receiving, and Marketing offices and had regular direct contact with frontline and other support staff.

### 3.4 Data collection and recording

Data were collected through semi-structured, in-person interviews, lasting approximately 60 min each. Interviews were conducted in a natural conversation style, initially establishing rapport, explaining the study and confirming principles of confidentiality and freedom to withdraw. Interview questions are depicted in Table 2 and focused on participants’ experiences of living and working during COVID-19 and how they coped during this time. Interviews were audio-recorded using a cell phone and transcribed by a professional transcriber. ATLAS.ti (ATLAS.ti Scientific Software Development GmbH, 2023) was used for data management and to support the organization of data analysis. All coding and themes were generated by the researcher and no AI-assisted coding features were used.

**Table 2:** Interview questions.

Question category	Question content
Opening question	Tell me about your experience of living and working since the start of the Covid-19 pandemic and the consequent lockdown in SA in March 2020
Deeper delve questions	What where your highs and lows during this time? Why? What effect did these experiences have on your work? What helped you to cope with your experience (probe organizational, collegial, personal resources).
Closing question	Is there anything else that you would like to add or share?

### 3.5 Data analysis

Thematic analysis (Braun et al., 2019; Braun and Clarke, 2012) was applied due to its suitability for large samples that are homogeneous in certain respects and heterogeneous in others (Spiers and Riley, 2019). The focus was on analysing data across data sets to reveal shared meanings rather than uncovering idiosyncratic or existential, ideographic meanings (Braun and Clarke, 2012; Spiers and Riley, 2019) while acknowledging the active interpretive role of the researcher in the interpretation process (Braun et al., 2019). As a flexible analytic method, thematic analysis accommodates both inductive (data-first) and deductive (theory-driven) reasoning (Braun et al., 2019; Braun and Clarke, 2012; Spiers and Riley, 2019), which was important in this study as codes and themes were initially directed mainly by data and the research aim. As the analytical process progressed, with iterative engagement across data sets, the researcher’s interpretive work became increasingly influenced by relevant resilience and SOC theory.

Thematic analysis entailed a recursive process of moving between and within six analytic phases (Braun et al., 2019). Firstly, to establish a deep connection and familiarity with the data, the researcher read and re-read the transcripts, while also listening to the audio recordings, and taking notes in the transcript margins in ATLAS.ti (ATLAS.ti Scientific Software Development GmbH, 2023). Secondly, the researcher identified interesting and relevant text sections in each transcription, rephrasing and condensing these sections of verbatim text into meaningful notes. Similar and recurring interpretations were further condensed, by constructing latent codes. A latent coding strategy refers to constructing deeper, implicit or conceptional meaning, from the condensed interpreted verbatim text (Braun et al., 2019). At this stage, data were transferred to MS Excel, creating tables with sections of text and corresponding interpretations. Thirdly, the researcher continued to categorize similar and overlapping codes into more comprehensive themes. The analytic trail from verbatim excerpt to condensed meaning to code, is depicted in the findings section for two sub-themes grouped under theme 2, in Tables 3, 4. During initial analytic phases 2 and 3, the codes and the initial themes were overwhelming and related to a wide variety of psychological constructs. To enhance clarity and focus, the researcher engaged with resilience literature, enabling a more focused alignment of codes to a refined set of resilience-related themes. The iterative work through phases 2 and 3 led to the fourth phase, namely organizing themes into a coherent thematic structure. In phase five, the researcher continued to move between data and theory to refine the conceptualization of each theme. The final phase entailed presenting the findings in a compelling and logical narrative related to the research aim.

### 3.6 Ethics and data quality

The study was reviewed and approved by the UNISA-CAES Health Research Ethics Committee (reference: 2020_CAES_HREC_139). The research organization gave written permission for the research. Potential participants were accessed via the HRM, who identified and contacted eligible employees and facilitated their connection with the researcher upon obtaining consent. Interviews were conducted in private offices to ensure confidentiality. Face-to-face interviews were allowed when lockdown restrictions were downgraded to levels one and two. Prescribed safety guidelines for research entailing human contact during alert levels one and two were followed, namely, appropriate screening procedures, wearing masks, sanitizing and maintaining a distance of at least two meters between researcher and participant. All participants gave written informed consent. It was confirmed that, if necessary, participants would also have access to the company’s employee assistance program. The transcriber signed a confidentiality agreement, and all files and recordings were password protected.

Data quality strategies focused on ensuring credibility, dependability, transferability and confirmability of the findings (Loh, 2013). Findings were supported with rich verbatim to enhance credibility. A thorough description of the research design, the sample and how thematic analysis was operationalized provides transparency and rigor, which promotes the study’s dependability. The transferability of findings to other retail organizations was pursued by providing a detailed background on the context of the study, including the organization and the sample. Grounding the findings in relevant psychological theory further supported confirmability.

## 4 Findings

The findings are structured along three core themes that describe organizational and management practices that (i) establish coherence, (ii) restore a sense of control, and (iii) nurture connectedness. The sub-themes provided insight into the informal and formal practices in the company. The thematic structure of the themes and sub-themes is depicted in Figure 2.

**Figure 2:**
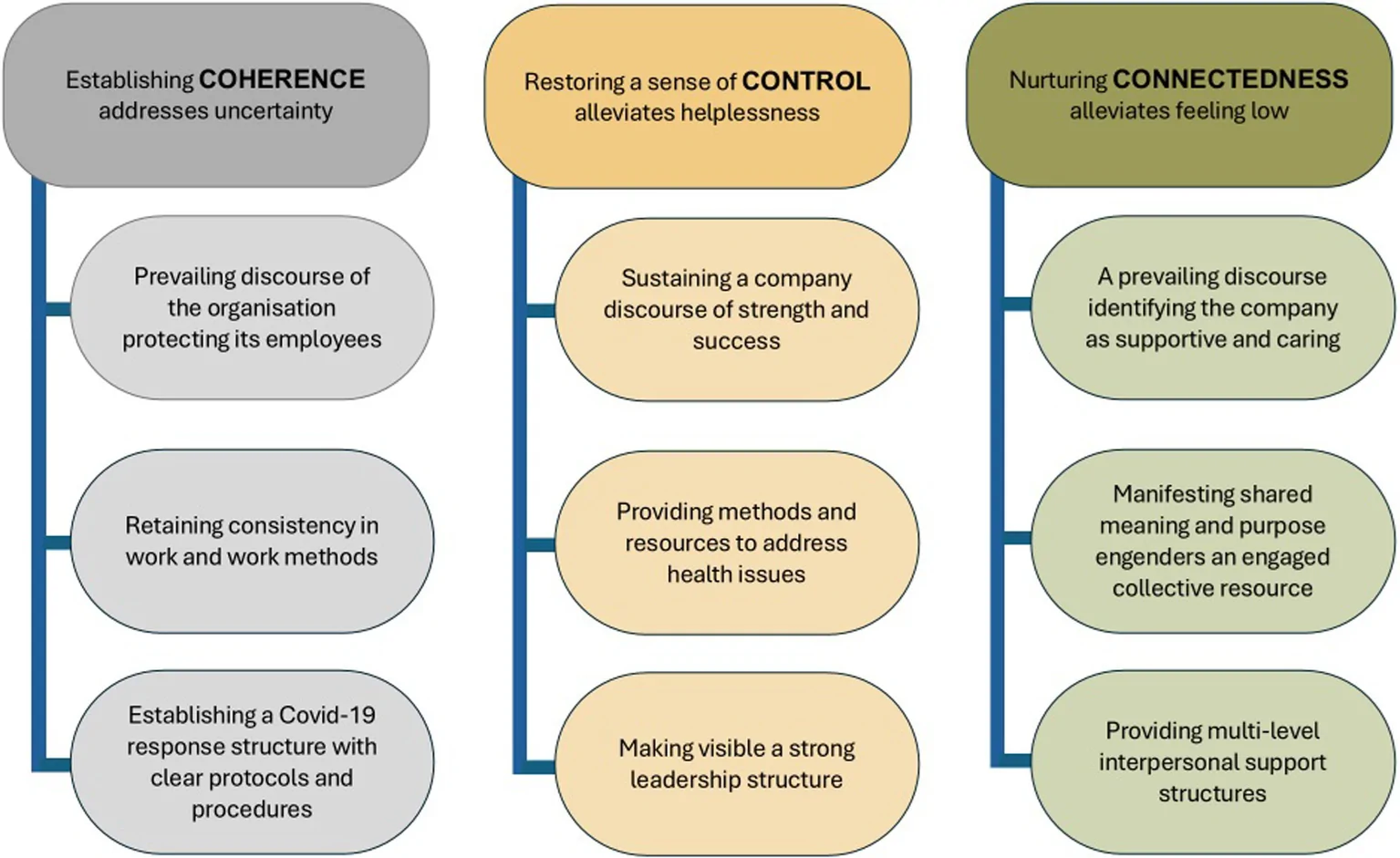
Thematic structure of the findings.

### 4.1 Theme 1: practices addressing uncertainty and confusion, establish coherence

Employee responses to the pandemic were characterized by uncertainty and confusion. For them, the situation lacked coherence, causing overwhelming fear. Certain organizational and management practices, however, helped employees feel that they could make sense of their situation. Such practices established coherence and became evident in (i) a prevailing discourse about the organization as protective of its employees, (ii) providing consistency in work and work method, and (iii) establishing clear protocols, procedures and policies.

#### 4.1.1 Prevailing discourse of the organization: protecting its employees

The company seemed to understand the importance of safety and job security as a measure of certainty during this extremely uncertain time. The managers shared their experiences of the company’s philosophy about and commitment to providing work, a salary and a safe work environment. W2P16 reflected, *“it really was a simple question of we need to balance keeping people safe and the business alive and going”*. W2P12 highlighted their primary focus during crisis planning, namely job security and safety:

*Thinking back to working in the first three weeks of lockdown, the scenario planning, and specifically planning around how we’re gonna keep everyone’s jobs… Our first priority was to protect our staff, so, whatever decision we made, it was to protect our staff. We run a business and if we protect our staff, we will trade… thereby ensuring that our staff have a job to come to, tomorrow morning and the next day and the year.*

Similarly, W2P14 explained commitment to employee safety and how this addressed fear and positively impacts employee coherence:

*People are afraid to die, and they think they’re gonna die, and then they don’t want to come to work… The steps that we put in place as a company for them to be safe, we hammered on that the whole time… Our values that we have, where the company is looking after the staff, that’s always the way we do things. Look after your staff first, because our thinking was if you look after staff and you can explain to them, you can come to work, it’s safe, then they will be at ease to help the customer… As days went by, people got more relaxed, because they start knowing what’s happening.*

The company’s concern for staff security alleviated employees’ fears of job loss and economic survival. W2P5, for example, observed: *“Our company… jo, they pay us normally that 21 days’ lockdown. When they called us and told us we need to come back to work, I was so relieved. I kissed my kids; Mummy’s still got a job”.* Others emphasized the company’s guarantees of job security and safety: *“Everything was being kept afloat, because thank goodness, [the company] was paying us in full. So that was one burden lifted off our shoulders, they gave us a guarantee that we are going to try, by all means, to keep all of you here”* (W1P7). Ultimately, employees felt protected by the company and, in turn, expressed understanding and commitment to the company: *“The director was angry, but you must think what he has to go through, his position in the company is extremely stressful because he protects his staff”* (W1P2). Similarly, W2P13 emphasizes: *“The company is protecting, our employees are protected”*.

The company’s commitment to job security and a safe working environment was an established norm, reconfirmed during the pandemic, which addressed key uncertainties and helped employees to cope with the overwhelming fear of survival.

#### 4.1.2 Retaining consistency in work and work methods

The company maintained work consistency by returning to regular working hours as soon as national regulations permitted. Then they kept work tasks and methods as consistent and familiar as possible. Being able to return to work supports coherence as it provides a sense of normality and a familiar focus (i.e., knowing what to do), as noted by W1P1:

*Thank goodness when they said we could return to work, just that bit of normality returned to people's lives. For me it was a high point during COVID to return to work permanently. I knew that I couldn't do nothing at home, I'll just feel out of control. I have control over my work. That kept me going, just to get back to work. To come back to work felt a bit normal.*

Returning to work also alleviated fears and uncertainties for W2P4, and for the first time since lockdown, she started to feel that the situation was making sense and that it was going to be fine: *“The [company name] was closed. That was tough and nobody knew what was going to happen. Luckily, we were blessed to get paid, and I was one of the first to go back to work. My manager phoned me and called me back. Then I was okay and thought this is fine”*.

After having to isolate, W2P6 noted that keeping work conditions as consistent as possible provided a sense of safety: *“When you came back to work, we still got our jobs, secure and safe the way they are, the same department, the same thing that you were doing”*. This consistency helped employees to replace despair and confusion with positive emotions such as hope and gratitude. W1P2 observed: *“We must be grateful for every little thing… we are very privileged at [company name]”.* Resuming their usual duties upon returning to work offered employees a sense of certainty rather than feeling incapacitated.

#### 4.1.3 Establishing a COVID-19 response structure with clear protocols and procedures

Management made and communicated decisions clearly to minimize uncertainty: *“Directors made quick decisions about who must do what, about hours and salaries and quickly communicated my responsibilities to me. It was clear what I had to do and when”* (W1P1). The data also showed evidence of the protocols and procedures that management implemented to address various workplace scenarios. W2P4 confirmed: *“Everyone was scared in the beginning, the managers were stressed that the shops will be closed, so they quickly put protocols in place to respond to any symptoms”*. This ensured certainty about how to respond and helped employees know what to do and whom to contact when they or any of their colleagues fell ill, how to handle customers and what to do at home. The participants highlighted how clear protocols removed uncertainty: *“It’s really easy to just follow the protocols”* (W2P13) and *“There’s some people whom I could tell were sick… so I just tell them, go home; go to the doctor and get tested, let me know. It’s easy for me to follow the right steps.”* (W1P8). In another example W2P6 referred to the leave policy: *“With our company, if you test positive for COVID, they give you that fourteen days to isolate yourself, that doesn’t affect your salary, they don’t deduct it from your leave days, it’s a COVID leave”*.

Management established a task team to develop, communicate and implement new protocols and procedures. Communication channels were established to convey decisions efficiently to all staff levels. The task team managed positive cases on a case-by-case basis. Integrated, holistic case management was ensured by tracking all the positive cases and potential risk cases across the company’s branches and business units. They set up a visible tracking board, which enabled them to know at any point in time how staff at different branches were affected. This integrated and holistic approach enabled strategically efficient managerial decision-making and empowered managers and supervisors to manage their areas of work proactively and effectively. As a senior manager and part of the task team, W2P12 provided insight into the sense-making value of the task team and the protocols and communication processes they put in place:

*We were quite busy putting protocols together and trying to make sense of how we are gonna deal with staff… It’s a lot of training and communication about the protocols, making sure everyone knows exactly what they are doing, why it is that they’re doing what they are doing. We would not have been able to manage it this well if we kept our decisions about protocols to ourselves. The aim was to tell the branch managers, they had to then tell their head of departments, and we made sure it filtered right down. I remember creating WhatsApp groups… here’s the protocol* [continues to explain protocols at length]*. So, I think it gave them comfort to know that there were plans and guidelines on how to stay safe*.

Apart from the task team’s communication about protocols, they also made progress-tracking visible to key role players. This supported risk management and provided a better sense of what was going on, as noted by W2P13:

*We have the board here, it just helps us track everyone, see where they are at. Obviously for the directors to make strategic decisions in terms of pockets of individuals being positive and looking after that department or looking after that branch. It just creates a better picture of where we need to put our focus.*

The protocols gave employees a sense of safety amidst the uncertainty and confusion: *“So, the company doesn’t allow us to come to work if somebody at home is positive, which is safe”* (W2P8). The protocols, task team and management thus established an instrumental structure that enhanced coherence. It ensured that managers had confidence, knowing how to manage situations and that employees had no doubt about what to do when confronted with potential or real infections.

### 4.2 Theme 2: practices alleviating helplessness restore a sense of control

During lockdown and upon returning to work, participants exhibited a sense of helplessness, accompanied by heightened anxiety. They perceived little control over what was happening. Yet company practices helped re-establish a sense of control by (i) maintaining a company discourse of strength and success, (ii) providing methods and resources for employees to manage their health, and (iii) demonstrating a strong leadership structure.

#### 4.2.1 Sustaining a company discourse of strength and success

Findings indicated that participants believed in the company’s strength and resourcefulness. They confirmed the company’s success and good management during the pandemic, identifying with the discourse that characterized the company as a successful and preferred employer under capable management. Table 3 provides a summary of the data supporting the company’s discourse of strength and success.

**Table 3:** Data analysis trail: sustaining a company discourse of strength and success.

P code	Verbatim excerpts – meaningful text	Condensed meaning	Latent code
W2P6	I can give a hand for management, it’s the way how they handled things… the pandemic never had an effect anywhere on our company. I do not know if it had an effect, but the company tried by all means to make sure that we stay positive and they continued making us happy	Business was well managed to ensure employee safety and satisfaction	Successful management of the business
W1P6	I think the way that [company name] manages it is good, the social distancing, the screening, customer screens at tills, ja it’s good management of things	Good management to ensure safety of people	
W2P16	We were well run, had a very calm influence from the directors, telling us do not worry if everything goes bad. So, the staff was very much calmed by that, knowing that we are not under the pressure that other companies are… as a company, we did very well, for two main reasons. Firstly, we were well guided, if I can put it like that. There was no serious pressure put on us to do this or do that or fend for yourself	Good management and leadership ensured people safety and appeased employees’ anxiety	
W2P13	We’re just very analytical and thorough in doing our investigations and making sure the right decisions are made, not just for the company, but for the employees as well… I think we did very well… it’s difficult to identify any particular high, I think just the way that we handled it.	Management approach to and decision-making during pandemic was effective	
W2P5	On my side, I think they did the best. They did. From the 21st of that March when they announced the lockdown, until now… they are doing the best, [company name] is the best, it is, I do not wanna lie, the best company to work for.	This company is the best employer	Excellent leadership and an outstandingly strong company
W1P10	I must say this, the directors are good, [name of a director] is the best	This company’s leadership is the best	
W2P12	We’re very blessed as a company that we are doing so well, and that we expanded, and we were able to give lots of people work… because we managed it so well, we haven’ had to close our stores…Bigger companies were not even close to where we are, you know, with knowing exactly what to do and each staff member knows what to do, and, you know, we are streets ahead of other corporates. Streets ahead, but because of that leadership, that’s the only reason we are there	The company is far better than its competitors because it managed the pandemic well and has good leadership	
W2P14	I think we did good. What we did compared to other companies, we were way in front of them, we are way better. I went to other companies as well to see how they do it, and it’s like if you walk in there and you watch them and they say they are not confident in what they are doing, they do not know what to do… They’re afraid. So, I think that’s where we were way ahead of other companies.	Believe the company is better than other companies and managed the pandemic much better than other companies	
W2P2	I think COVID evolves, and we must evolve with it. So far with us at work I think we were good, we were well off.	Confidence in the success of the company despite the pandemic	Strong and successful company
W2P14	The name of the company was good, we did not worry about the company name, it was good	Confidence in the good reputation of the company	
W2P4	It was from nothing to a masterpiece. I mean his own words were ‘from bad to best’… I’m the humblest person, that gubbly child who does not know what to say to the big bosses, but I do my work with pride.	Taking pride in work and aligning with the high standards of the company	

#### 4.2.2 Providing methods and resources to address health issues

The company alleviated survival anxiety by providing tangible methods and resources for employees to manage symptoms of illness, making them feel less helpless when having to manage their health: *“The whole shop is full of sanitizer, we spray in the mornings, we measure our temperature, we did all those things at work”* (W1P3). Tangible resources regarding access to testing and covering testing expenses were provided: *“The company, they pay for us to go and get tested, it’s one thing that I’m clapping hands for, because at that time it was difficult, you must queue at the public clinic, you can only get your result after five days”* (W2P5). This was confirmed by W2P8: *“The company they took you to their doctor, at their own expense”*. Employees were assisted with administration around testing as well: *“They said I must go for a test, then [name] said, ‘Okay, I’m just going to prepare paperwork, you just wait for me in your car, then I’ll bring the paperwork for you’”* (W1P10); and *“My manager asked me ‘Can you drive now and come here to work to get the necessary papers and to go to the company’s doctor”* (W2P10).

The methods and resources provided to address employee health concerns were instrumental in making them feel contained. When they felt physically sick, mentally confused and scared, they did not need to worry about how to manage the situation or where to access health resources. A participant recalls: *“When I arrived at work, I was very tired. Luckily, there’s some people that are scanning us outside. I called one of them and I sat down, because I was very tired, I don’t know what’s happening to me. Maybe after a few minutes, he said, go and see the doctor, then we’ll communicate with you on the phone”* (W1P5). When people exhibited symptoms, they felt incapacitated by fear, but the company’s pragmatic approach in this regard brought much-needed emotional relief and a sense that things were under control and manageable.

#### 4.2.3 Making visible a strong leadership structure

A strong leadership structure was visible throughout the organization. It manifested in the task team’s and senior managers’ regular communication, as well as frequent in-person contact with employees. This made the task team and managers accessible to employees, confirming that things were under control and alleviating their sense of helplessness. Table 4 summarizes the supporting data.

**Table 4:** Data analysis trail: making visible a strong leadership structure.

P code	Verbatim excerpt – meaningful text	Condensed meaning	Latent code
W2P14	We had a good task team, I do not know how we put it together, it was just quickly. The one night we said task team, and we phoned in and it just gelled… It was a very good task team and we worked together.	Establishing a task team	Well-functioning task team amplifies leadership direction throughout the company
W2P15	The task team that she established worked on a checklist, so we worked every day on ticking off certain aspects that had to be covered and that was mostly informed by what was given through by the task team from head office. They approached it by monitoring and diagnosing on a continuous basis.	Task team gave direction to management on branch level	
W2P12	It wasn’t a once-a-week meeting, and you must just carry on, on your own. There’s a task team meeting every day, and these are the decisions we have now made… So, the leadership made a huge difference… I think for any crisis, or any pandemic going forward, there’s strong leadership needed and strong direction and liaising literally daily that people know exactly what’s happening.	Task team provided leadership, direction and effective management through regularly established communication channels	
W2P12	We would not have been able to manage it this well if we kept our decisions about protocols to ourselves. The aim was really to tell the branch managers, they must then tell their heads of departments, and we made sure that it filtered down, right down to the lowest level.	Establishing a structured line of communication right through company	Well-established, real-time, supportive communication structure throughout the company
W2P16	There was very good interaction between the directors and management at each branch… guidance from the directors was good and we were told it does not matter what call you make; we have got your back.	Open and supportive communication line between levels of management	
W2P13	There’s one director allocated to a branch, and you spoke to him, or one body at head office allocated to a branch that you could speak to.	Established leadership structure that made leaders accessible throughout the company	Leadership structure is accessible to all
W2P1	There was communication. A WhatsApp group was set up with [manager] and he asked us every day about our symptoms and stuff, and I think that was very good for us who had been positive and those who did not have it.	Leaders establish direct communication channels	
W2P4	I’m being appreciated for what I do. Management and head office saw me, recognized me. I mean [name] was there the other day and [name], and [name] and [name] were there, they all came to have a look.	Leaders are on the floor, connecting with employees	Visible, accessible and reliable leadership structure present throughout company
W2P11	Managers were sending us the messages, like the support messages. They say please, please, please staff sanitize, take care of yourself, this thing is here.	Employees feel in touch with leaders through management’s direct and regular communication	
W2P3	A high point for me was when the director came to the branch 1 day and talked to me. He saw I was down in the dumps because our landlord wanted to put us out. And the director made arrangements for the work to assist us… and we could move forward again, but if it wasn’t for the director coming to the branch…	Leadership presence and accessibility enabled manageability.	
W2P2	The boss feels very strongly about visiting the branches and still goes whenever he can. The personal connection is important, to get to the branches and have one-on-one interactions with people.	Leadership presence on shop floor to personally connect with staff.	
W2P15	She really showed her face on the floor a lot. It was important for her to go out and to connect with the staff members, on a regular basis, asking them how are they doing? Is there anything that she can help them with?		

### 4.3 Theme 3: practices alleviating feeling low, nurture connectedness

The pandemic left participants feeling isolated and alone, causing them to feel disheartened. Company practices that played a pivotal role in alleviating low mood included (i) portraying the company as supportive and caring, (ii) communicating shared meaning and purpose, and (iii) having multiple interpersonal support structures throughout the organization. These practices seem to revolve around re-establishing a sense of belonging or connectedness.

#### 4.3.1 A prevailing discourse identifying the company as supportive and caring

There was a strong prevailing discourse among participants about being cared for in and by the company. There was also closeness among colleagues who felt that they were seen and known by others in the workplace, reinforcing a sense of belonging: *“I just talked with my best friend at work first before I go to other people, and she talked to the other cashiers, so they come to [support] me, people come next to me.”* (W2P10). W1P10 referred to strong connectedness in the workplace by talking about colleagues in familial and spiritual terms: *“There’s this one friend at work. God blessed me with her. God said, this is your sister, take care of her, she will take care of you, and I’m sorted, I’ve got friends, I’ve got lovely colleagues”*. W1P1 expressed the bond she experienced with co-workers: “*It was nice coming back to work, just to see my people again. Colleagues saying, ‘Welcome back, it’s nice to see you here again*’”. W1P10 even expressed personal attachment to management:

*My management, I don’t know how they do it, but I remember getting a call from [name]. [Managers’ names] were always there for me, and now I must fight this, I will be fine. They're like ‘anything you need, just shout’. The only reason people give up on life, it’s a support system, if you don’t have it, you’re all alone, but I'm blessed to have people that actually care.*

Referring positively to colleagues and management exemplified the participants’ sense of belonging and connectedness to the company. W2P10 emphasized: *“Management they were giving us support, support for the staff to show that they really care. You must look after each other, like families”*. W2P15 similarly referred to a senior manager in the following way: *“People come to her, see her as their mother, being involved and creating a sense that she cares and that they are really valuable to her”*. The prevailing discourse identified the organization as a caring family. W2P5 summarized: *“I wanna thank this company. They did the best, they did everything they could… You know when they say [name of company], it’s not a workplace, it’s more like family, it’s true”*.

#### 4.3.2 Manifesting shared meaning and purpose engenders an engaged collective resource

The company successfully conveyed a shared meaning and purpose. This was demonstrated in how employees share in the company’s commitment to business survival and keeping customers safe and happy, as well as expressing emotions such as pride and gratitude toward the organization. W2P12 noted: *“I think the high, almost on a daily basis is being proud of the task team and proud to work for a company who has made this a priority”*. Similarly, W2P4 observed:

*In the past I think I took work for granted, some days getting up and not feeling up for work, but now I’m more grateful for what I have. I take pride in my work, I take honor in my work and I do it to help the company.*

Shared meaning and purpose are motivational, as confirmed by W1P10’s expression of agency and commitment: *“I’m just going to do my part to keep myself and my family and the people that I love and my colleagues safe”*. W1P1 also emphasized her commitment: *“I mean this is my department, give it to me, it doesn’t matter what we are going to do, I promise you I will do it”*. Noting his close connection with the company, W1P5 believed that by surviving, he is helping the company to survive: *“At my workplace, they are giving me very good support… My voice was not fine, but I promised them that I’ll make it. I was praying, yes, let us make it”*. The strong connection between staff and management inspired productivity and efficiency, and according to W1P2, ultimately ensured the shared success of the company:

*We just went from strength to strength. But it's also the staff's attitude and it comes from management. The staff could just as well stay at home or just not help the customers, but it's as if the staff forget about that when they are in the store working with the customers. It's stuff like that what makes it easy and worthwhile to work.*

W2P13 confirmed that the company’s success depended on collective commitment:

*The business just grew and grew since last year and everyone kept to what is expected and maintained their heads in having to know that they’ve got a job to do, with the stresses that they experience at home and obviously having to deal with the fact that they might get exposed to COVID, the majority of the staff did exactly what was expected and worked as a team.*

Collective meaning and purpose provided employees with emotional resources they could rely on to remain resilient and motivated.

#### 4.3.3 Providing multi-level interpersonal support structures

As previously mentioned, various formal and informal support structures were made available to employees across the company. This included management support, HR support, collegial support and task team support. W1P9 observed: “*Everyone at work was supportive”* and W2P10 confirmed: “*There’s a lot of support from managers and colleagues*”. Further examples provided evidence of the personal support received from formal protocols and management methods and speak to the authentic informal support that employees received. It gave employees a strong sense of comfort, for example when W1P5 who upon testing positive and in a panicked state, received a call from HR: “*It was the HR, he said calm down, calm down, it’s not the death sentence. He talked to me; he speak to me*”. This support also gave W1P10 strength: “*What gave me strength, it’s the love that I got from work… you know they actually care about me. Ja, that’s the only thing that helped me*”. Similarly, W2P5 was provided with hope and a sense of belonging when falling ill with COVID-19:

*The boss was supportive. She would call you and ask you, ‘How are you feeling today?’, and the one from head office, she was also like those people who made you feel like, you know what, I’m gonna, get through this, I’m gonna be okay… Managers will call you each and every day, asking how are you feeling. They will send you get-well messages, they motivate you, and they make you feel like you’re not alone.*

The support structures culminated in employees feeling connected and worthy, as W1P7 noted: *“My support system was amazing. Work, they would call, [name of colleague] would phone me, [another name of colleague] would phone me… At no point did I feel like I was doing it alone”*. In another example, W2P9 testified: *“They used to check me every day. They used to call me, WhatsApp me, and they just offered me everything that I needed”*.

In the task team and various management teams across the company, colleagues felt supported by receiving regular contact. Ultimately, the personal nature of support and the direct involvement of management were pivotal in containing overwhelming anxiety and curbing demotivation.

## 5 Discussion

Taking a salutogenic perspective, this study explored organizational and management practices in support of retail employees’ coping and resilience during the COVID-19 pandemic. The findings articulate practices that enhance coherence, control, and connectedness by addressing uncertainty, helplessness and low mood, and thereby fostering resilience within the organization. The same practices invariably increase employees’ SOC, strengthening their comprehensibility, manageability and meaningfulness. Firstly, the company addressed uncertainty and confusion through practices that establish coherence in the work context, such as maintaining discourse about the company as protective of its people, retaining work consistency, and establishing clear COVID-19 response structures, protocols and procedures. A clear response structure corresponds with research recommendations emphasizing the implementation of relevant COVID-19 safety protocols, procedures and guidelines in the retail sector to curb employee distress (Choi and Marinescu, 2025; Mayer et al., 2022; Janson et al., 2021; May et al., 2021). The result was a workforce feeling safe, knowing what to expect and do at work and being clear about how to respond to pandemic-related threats and eventualities. Coherence practices thus enhanced comprehensibility, namely, employees’ ability to make sense of the pandemic and to think and react rationally in unknown circumstances.

Secondly, it was found that company practices helped restore a sense of control. This was achieved by sustaining a discourse of strength and success, providing methods and resources to address pandemic-related health concerns and demonstrating a strong leadership structure. Employees reaffirmed perceptions of the organization as strong and resourceful. The availability of healthcare resources and the establishment of socio-economic stability further enhanced their sense that the situation was manageable. These findings underscore the importance of the organization establishing and communicating job security as soon as possible (Ghani et al., 2022) and providing financial support to mitigate economic harm (Ashforth, 2020; May et al., 2021) during crises such as COVID-19. Effective leadership serves as a crucial psychological resource, strengthening employees’ confidence that reliable individuals are available to navigate current and future predicaments. The need for supportive leadership has been alluded to in COVID-19 studies highlighting the negative effect of poor relations with managers and supervisors, on employee well-being (Choi and Marinescu, 2025; Gaan and Shin, 2023). Practices aimed at alleviating helplessness were thus consolidated as those restoring control and supporting employees’ manageability, by strengthening their confidence in having the necessary resources to cope with pandemic-related stressors.

Thirdly, the company addressed employees’ feelings of isolation and diminished morale by implementing practices that responded to their fundamental need for connection. Practices nurturing connectedness included promoting a discourse that identifies the company as supportive and caring, establishing shared meaning and purpose in the workforce, and providing multi-level interpersonal support structures. These practices strengthened employees’ sense of belonging and purpose, encouraging emotional positivity and commitment to their work. Nurturing connectedness aligns with COVID-19 studies underscoring the value of social support (Ashforth, 2020), relationships and collectivism, for promoting employee well-being (Gaan and Shin, 2023; Ahmed and Akaak, 2024).

Integrating the findings with the theoretical framework presented in Figure 1 results in a salutogenic framework for managing disruptive change as depicted in Figure 3. The salutogenic framework vertically categorizes management and support practices into Reich’s (2006) 3-C framework, and aligns with how each practice category (coherence, control and connectedness) supports the three SOC sub-components. Horizontally the salotugenic practices follow three tiers which are further categorized into *tangible* and *covert* practices. On the bottom and middle tiers, the organization established tangible practices, namely (i) *structuring* (i.e., providing a COVID-19 response structure, a visible leadership structure and multi-level support structures), and (ii) *methods and resources* (i.e., providing work consistency, methods and resources to address health concerns, and a shared meaning and purpose). Covert practices are indicated in the top tier in Figure 3 and entail practicing sustained discourse about the organization being protective, strong and successful, and caring. Covert practices thus revolve around maintaining an *organizational identity*, with which employees strongly and positively identify.

**Figure 3:**
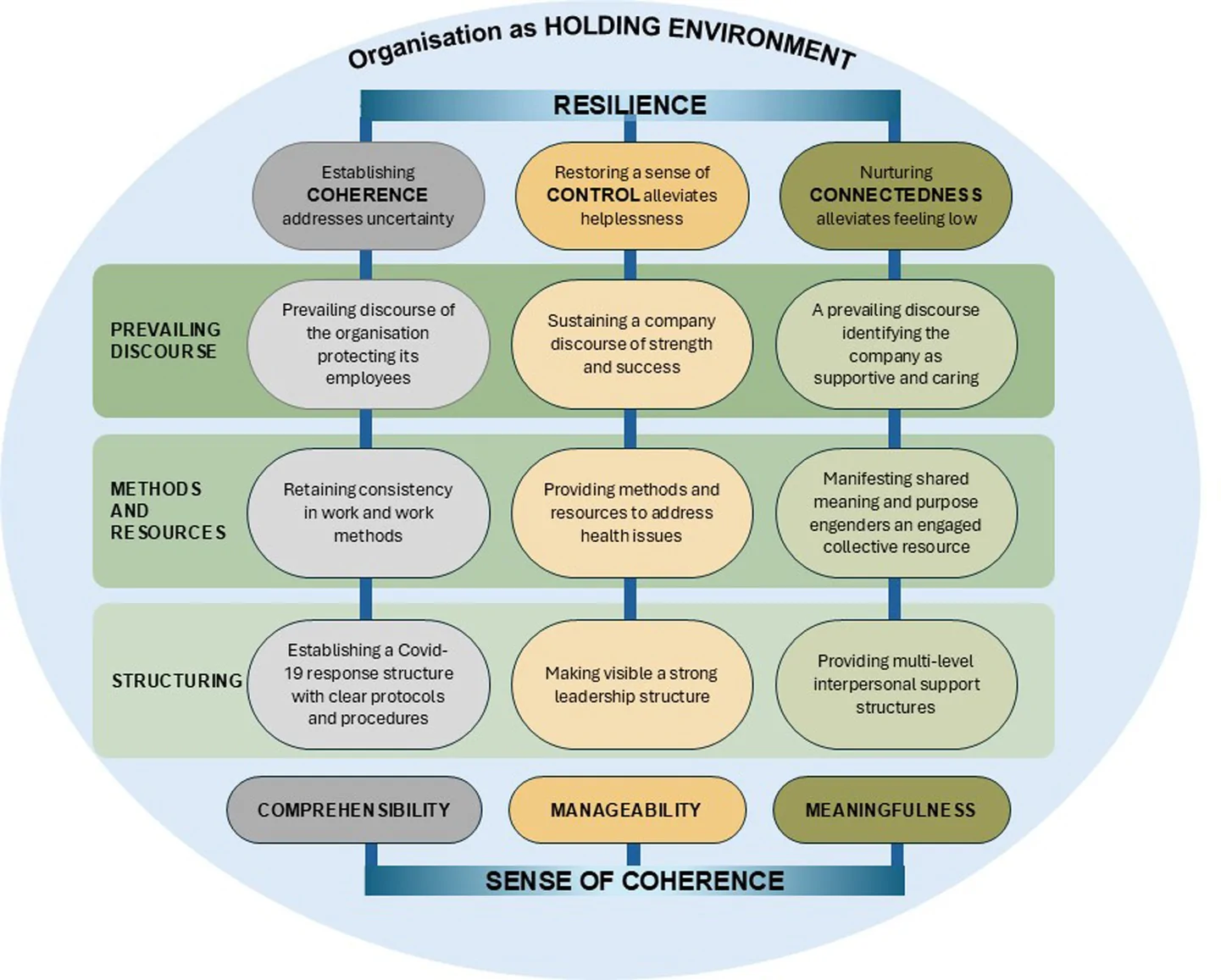
A salutogenic framework for managing disruptive change.

Ultimately, the salutogenic framework proposes the organization as a holding environment, which is essential when employees experience disabling anxiety at work (Kahn, 2001). A holding environment reduces disturbing emotions and facilitates sense-making (Petriglieri and Petriglieri, 2010). The proposition constructed in this study suggests that an organization seems best positioned to foster resilience when it acts as a holding environment that engages SOC-supporting practices on both tangible and covert levels. Covert salutogenic practices support organizational identification (cf. Ashforth, 2020), a deep-seated, value-driven identity construction process related to the emotional reality of the organization (Armstrong, 2005) and to important stabilizing work identity dynamics (Brown, 2017). Organizational identification enables coping and commitment (Ashforth, 2020) and offers a collective defense against anxiety (Brown, 2017) or “organization-in-the-mind” that helps to manage vulnerability and anxiety in response to disruptive change (Armstrong, 2005). Tangible salutogenic practices must be implemented to reinforce organizational identity as such practices signal the real or lived nature of its underlying values (Ashforth, 2020). Tangible practices include consistent and clear work methods, as well as offering a range of accessible health and safety resources. Crisis response systems, robust leadership structure, and additional interpersonal support structures are needed to contain emotional disruption and guide constructive behavioral responses.

## 6 Conclusion

This study set out to address a gap in research about management and organizational practices that foster resilience during times marked by disruptive change. It was conducted during the height of the COVID-19 pandemic, engaging retail employees who were particularly vulnerable to anxiety and stress at this time and are central to socio-economic stability and growth (Benchekroun et al., 2022). The study was guided by salutogenic theory as recent COVID-19 research highlights the critical role of enhancing employees’ resilience (Mayer et al., 2021; Ngoc Su et al., 2021) and SOC (Barni et al., 2020; Mana et al., 2021) in mitigating the pandemic’s adverse psychological impact on vulnerable frontline employees. In addressing the unique plight of retail workers as unexpected frontline employees, this study proposes a pragmatic salutogenic approach to how they can be managed for sustained resilience and productivity during a crisis.

The study contributes to salutogenic theory, explicitly aligning resilience in the organization with SOC, confirming the utility of the 3-Cs resilience framework during actual crises and suggesting salutogenic practices in a unified framework. Aligning resilience with SOC is useful because resilience remains broadly conceptualized with little agreement on its sub-components, resulting in inconsistent measurement (Kase et al., 2024; Smuda-Kocoń, 2024). On the other hand, SOC is a clearly defined, well operationalized and measurable construct (Moksnes, 2021) with stronger predictive validity for well-being outcomes compared to resilience (Kase et al., 2024; Keil et al., 2017). To strengthen resilience, research confirms that organizations should focus on work environments that enable employees’ SOC (Mayer et al., 2021). Existing studies however focus on job resources intended to enhance SOC, while paying comparatively less attention to organizational and management practices that contribute to SOC development (Broetje et al., 2020; Jenny et al., 2022). The salutogenic framework that was constructed through this study encompasses practices that support employees’ SOC on both covert and tangible levels, which organizations can follow to foster resilience.

The findings support the usefulness of the 3-C’s resilience framework conceptualized by Reich (2006), by offering examples of how these principles can be operationalized to foster organizational resilience during an actual crisis. Psychological resilience is typically regarded as an individual trait, characteristic or skill that can be optimized in the work context through training, employee assistance programs or counseling services (Asthana et al., 2024). This study rather offers a systems perspective to resilience in providing exemplary organizational and management practices fostering organizational resilience. In so doing, the study contributes to creating what Roskams and Haynes (2020) conceptually refers to as a salutogenic workplace design—synthesizing organizational and management practices that foster resilience into a pragmatic salutogenic framework.

The study presents some limitations. First, although the research was confined to one organization, data were collected from participants in various roles, branches and hierarchical levels, enabling rich understanding of the way the crisis was managed. Some differences in experience were apparent between non-managerial and managerial participants. Non-managerial participants predominantly expressed anxiety about their own health and that of loved ones, as well as job and financial concerns. They consequently looked to the organization for protection, continued employment, income and health related support. Managers, in contrast, expressed greater concern about containing virus transmission, safeguarding employees and customers, and ensuring the operational and financial survival of the business. No clear patterns distinguishing experiences between participants were further noted or deeper explored, which may be regarded as a second limitation. However, the study did not aim to conduct subgroup comparisons and participant heterogeneity was intended to enhance the richness and breadth of the data. Management accounts provided insight into organizational practices and strategies implemented during the time, while employee accounts illuminated how these practices were experienced, enabling a synthesis of these accounts into an integrated framework for guiding organizational and management practices during crises. Thirdly, the preconceived theoretical approach led to one way of conceiving the findings, which may differ when taking another theoretical stance. This is a relevant limitation, given that the conclusions were structured thematically using a specifically defined resilience and SOC framework. However, findings derived from hermeneutic phenomenological studies are not presented as conclusive and irrefutable facts, but rather as insights that enrich and expand the relevant knowledge base (Alsaigh and Coyne, 2021). Being guided by an appropriate theoretical perspective helped in constructing and verifying the conceptual insights and in organizing the findings into a meaningful thematic structure. The unique family-owned context of the study constitutes another potential limitation to transferability, as the participating organization was resource-rich and exceptionally supportive. Relying on the HRM as gatekeeper for participant referrals may also have introduced recruitment bias, potentially resulting in selecting participants with a favorable employer perspective. While these aspects may have contributed to the notable positive employee experiences, it also allowed for the identification of resilience-promoting practices that align with the study’s salutogenic focus. It is important to acknowledge that participants’ narratives reflected pervasive fear, uncertainty and survival anxiety. Negative experiences revolved around fearing the virus and loss of life (their own and of their loved ones); anger and uncertainty were directed toward government and law enforcement services; work-related challenges included transport difficulties and uncertainties about coming back to work after lock-down and isolation. The absence of negative organizational perceptions in the data may be attributed to the organization as a source of certainty and support during a time of illness, isolation and heightened fear. The organization provided certainty when they were uncertain; stability in a time when others around them lost their work and income, and meaning in having something to do and somewhere to belong. The uniqueness of the study primarily stems from its timing. Interviews were conducted when emotional experiences were fresh in the mind, yet participants had developed sufficient reflective capacity to articulate the organizational factors that supported their coping. In this way, the study capitalized on utilizing the COVID-19 pandemic as a poignant example for fostering resilience in organizations during adverse times.

Further research is needed to operationalize the 3-Cs framework as a measure of organizational resilience. The findings highlighted the practical importance of SOC as a resilience resource and advanced the notion of the organization as a holding environment to support SOC and build organizational resilience. Post-COVID-19 research is recommended to confirm the propositions made here, specifically in terms of assessing the long-term effects of the practice guidelines within the proposed salutogenic framework on employee and organizational resilience.

## Data Availability

The datasets are not publicly available to safeguard confidentiality and anonymity of the research organization. Permission for public data sharing was not obtained because sharing full transcripts would disclose contextual information that could compromise the anonymity of the participating organization and its employees. Nevertheless, extensive verbatim quotations are presented in the findings section to substantiate interpretations. Requests to access the datasets should be directed to barnaha@unisa.ac.za.
